# 

**Published:** 1887-05

**Authors:** 


					﻿io cts. a copy
MAY, 1887.
$i.oo per year.
HALLS
eJ0VKN/\LJ
HEALTH
ESTABLISHED 1854
U°i- 34
c/l/2.- 5.
OFFICE: 206 BROADWAY, EVENING POST BUILDING, Boom 11. N. T.
Entered as Second-class Matter at the Post-Office, New York.
				

